# Identification of two chickpea multidrug and toxic compound extrusion transporter genes transcriptionally upregulated upon aluminum treatment in root tips

**DOI:** 10.3389/fpls.2022.909045

**Published:** 2022-08-05

**Authors:** Yong Jia, Karthika Pradeep, Wendy H. Vance, Xia Zhang, Brayden Weir, Hongru Wei, Zhiwei Deng, Yujuan Zhang, Xuexin Xu, Changxing Zhao, Jens D. Berger, Richard William Bell, Chengdao Li

**Affiliations:** ^1^Western Crop Genetic Alliance, Murdoch University, Perth, WA, Australia; ^2^State Agricultural Biotechnology Centre, College of Science, Health, Engineering and Education, Murdoch University, Perth, WA, Australia; ^3^Department of Primary Industry and Regional Development, Government of Western Australia, Perth, WA, Australia; ^4^Centre for Sustainable Farming Systems, Future Foods Institute, Murdoch University, Perth, WA, Australia; ^5^Shandong Provincial Key Laboratory of Dryland Farming Technology, College of Agronomy, Qingdao Agricultural University, Qingdao, China; ^6^College of Horticulture, Qingdao Agricultural University, Qingdao, China; ^7^Agriculture and Food, CSIRO, Floreat, WA, Australia

**Keywords:** aluminum tolerance, chickpea, wild *Cicer*, hydroponics, multidrug and toxic compound extrusion, malonic acid, protein modelling, root elongation

## Abstract

Aluminum (Al) toxicity poses a significant challenge for the yield improvement of chickpea, which is an economically important legume crop with high nutritional value in human diets. The genetic basis of Al-tolerance in chickpea remains unclear. Here, we assessed the Al-tolerance of 8 wild *Cicer* and one cultivated chickpea (PBA Pistol) accessions by measuring the root elongation in solution culture under control (0 μM Al^3+^) and Al treatments (15, 30 μM Al^3+^). Compared to PBA Pistol, the wild *Cicer* accessions displayed both tolerant and sensitive phenotypes, supporting wild *Cicer* as a potential genetic pool for Al-tolerance improvement. To identify potential genes related to Al-tolerance in chickpea, genome-wide screening of multidrug and toxic compound extrusion (MATE) encoding genes was performed. Fifty-six *MATE* genes were identified in total, which can be divided into 4 major phylogenetic groups. Four chickpea *MATE* genes (*CaMATE1-4*) were clustered with the previously characterized citrate transporters *MtMATE66* and *MtMATE69* in *Medicago truncatula*. Transcriptome data showed that *CaMATE1-4* have diverse expression profiles, with *CaMATE2* being root-specific. qRT-PCR analyses confirmed that *CaMATE2* and *CaMATE4* were highly expressed in root tips and were up-regulated upon Al treatment in all chickpea lines. Further measurement of carboxylic acids showed that malonic acid, instead of malate or citrate, is the major extruded acid by *Cicer* spp. root. Protein structural modeling analyses revealed that CaMATE2 has a divergent substrate-binding cavity from *Arabidopsis* AtFRD3, which may explain the different acid-secretion profile for chickpea. Pangenome survey showed that *CaMATE1-4* have much higher genetic diversity in wild *Cicer* than that in cultivated chickpea. This first identification of *CaMATE2* and *CaMATE4* responsive to Al^3+^ treatment in *Cicer* paves the way for future functional characterization of MATE genes in *Cicer* spp., and to facilitate future design of gene-specific markers for Al-tolerant line selection in chickpea breeding programs.

## Introduction

Chickpea (*Cicer arietinum* L.) is a valued grain legume worldwide, ranking second in area but third in production after soybean and dry bean (Rawal and Navarro, 2019). Chickpea seed is rich in protein, minerals, vitamins, and fiber, which provides many health benefits in diets (Wallace et al., 2016), thus playing a critical role in human nutritional security. Over 60% of world chickpea production is from India, whilst Australia, Canada, and Argentina have seen increasing chickpea production in recent years, and become leading chickpea exporters (Merga and Haji, 2019). During the past two decades, the world production of chickpea has increased steadily from ∼7 million tons to ∼14.5 million tons (Rawal and Navarro, 2019). However, chickpea yield has remained relatively stagnant.

Low pH and Aluminum (Al) toxicity has been recognized as major soil constraints for crop production. Around 30∼40% of the arable soils in the world are acid soils, and the area and severity continues to increase due to factors such as acid rain, intensive agriculture, and the continued application of ammonium-based nitrogen fertilizers (Zheng, 2010). The toxic Al^3+^ species becomes soluble at low pH which inhibits root elongation, thereby impairing nutrient and water uptake, leading to crop yield loss. In chickpea, Al stress can inhibit root growth, as well as nodulation and nitrogen fixation (Singh et al., 2012; Choudhury and Sharma, 2014; Vance et al., 2021). In India (Mandal et al., 2019) and Australia (de Caritat et al., 2011), acidic soils account for a large proportion of the arable land. Thus, improved Al tolerance within chickpea cultivars would lead to higher crop yield on acid soils and the possibility of expanding chickpea production on soils where Al toxicity currently hampers cultivation.

Plants have developed various mechanisms to alleviate Al toxicity under acidic soils. The major mechanism is through the Al-activated release of carboxylic acids from root tips (Bojorquez-Quintal et al., 2017). In barley, Al tolerance is achieved by the Al-induced secretion of citrate from roots, which chelates the toxic Al^3+^ in acidic soils (Fujii et al., 2012). The secretion of citrate is facilitated by the *HvAACT1* (Al-activated citrate transporter) gene encoding an enzyme in the multidrug and toxic compound extrusion (MATE) family (Furukawa et al., 2007; Fujii et al., 2012). MATE transporters occur widely in nature, transporting substrates such as organic acids, plant hormones and secondary metabolites in both prokaryotes and eukaryotes (Takanashi et al., 2014). Homologous MATE proteins with similar citrate transport functions have been identified from wheat (Tovkach et al., 2013), maize (Maron et al., 2010), sorghum (Magalhaes et al., 2007), rice (Yokosho et al., 2009), *Medicago truncatula* (Wang et al., 2017), and *Arabidopsis* (Durrett et al., 2007). In addition to the citrate transporter MATE, an Al-activated malate transporter (ALMT) has also been reported in many plants and is associated with the malate-mediated Al detoxification (Sasaki et al., 2004; Zhang et al., 2019). Genetic studies on the Al-tolerance mechanism in grain legumes are still very limited. Several transcriptome analyses in root tips of legume plants indicated that MATE encoding genes are transcriptionally responsive to Al treatment, and may have a similar Al-tolerance function (Chandran et al., 2008; You et al., 2011; Liu et al., 2016).

In chickpea, the genetic basis of Al-tolerance remains obscure. Preliminary investigations have indicated that acid tolerance variations are present across different genotypes (Rai, 1991; Manorma Sharma et al., 2015). Using two genotypes of varying Al-tolerance, Singh and Raje (2011) showed that Al-tolerance in chickpea may be controlled by a single dominant gene. However, no candidate gene has been identified to date. Furthermore, the current chickpea cultivars contains limited genetic variation related to biotic and abiotic stressors (Berger, 2006; Singh et al., 2008), which hinders the breeding progress for higher chickpea grain yield. The wild progenitor of chickpea (*Cicer reticulatum*) and its close relative, *C. echinospermum*, provide diverse gene pools for chickpea improvement that was recently widened by collection throughout SE Anatolia, Turkey where sampling covered a wide range of locations, climates and soil types (von Wettberg et al., 2018). Interestingly, these two wild relatives of chickpea are found in different soil types: biologically derived limestone and sandstone soils for the former contrasting with basaltic soils for the latter. Collection sites differ in terms of climate and soil properties: *C. reticulatum* collection site soils were more fertile and more alkaline than those where *C. echinospermum* was collected (von Wettberg et al., 2018). Most importantly, *C. reticulatum* and *C. echinospermum* have no reproductive barrier with domesticated chickpea, therefore trait diversity in these wild *Cicer* spp. can be readily introduced in chickpea breeding programs (Singh et al., 2008).

In this study, we aim to explore the Al-tolerance variation within wild *Cicer* species, and identify the potential candidate genes contributing to Al-tolerance. Selected wild *Cicer* accessions were assessed for Al-tolerance based on root elongation measurements. Genome-wide survey, phylogeny, and transcriptional analyses of the MATE gene family in chickpea were performed. Root-secreted carboxylic acid extrusion was also determined. Protein structural modeling and substrate docking were performed to explain the carboxylic acid profile. The chickpea pangenome data was also searched to assess the genetic diversity of wild and cultivated chickpea. This study is the first report of MATE-encoding genes transcriptionally associated with Al treatment in wild *Cicer* root tips, facilitating the future design of gene-specific markers for improved Al-tolerance in chickpea breeding programs.

## Materials and methods

### Plant materials, hydroponic cultivation, and tissue sampling

One chickpea cultivar (PBA Striker) and 8 wild *Cicer* lines were included in low pH ± Al hydroponics screening (5 *C. reticulatum*: Bari2_074, CudiB_008B, Kayat_064, Sarik_067, and Sarik_073 and 3 *C. echinospermum*: Deste_063, Deste_064 and Karab_062 obtained from the germplasm collected from southeastern Anatolia, Turkey). Around 50 seeds for each line were used. Sterilized seeds (3% sodium hypochlorite for 5 min, followed by rinsing 5 times with de-ionized water) were placed on a petri-dish covered with wet paper towel to allow germination for 4 days at 22°C in a growth chamber.

On day 5, seedlings were transferred to 5-liter containers containing control solution with constant aeration. All seedlings were initially in the control condition. The same nutrient solution at pH 4.2 contained (μM) was used as in a previous study (Vance et al., 2021): KNO_3_, 650; CaCl_2_.2H_2_O, 400; MgCl_2_.6H_2_O, 250; NH_4_NO_3_, 40; H_3_BO_3_, 23; (NH_4_)_2_SO_4_, 10; Na_2_HPO_4_, 5; MnCl_2_.4H_2_0, 9; ZnSO_4_.7H_2_O, 0.8; CuSO_4_.5H_2_O, 0.3; Na_2_MoO_4_.2H_2_O, 0.1. Iron (20 μM) was supplied as Fe-EDTA prepared from equimolar amounts of FeCl_3_.6H_2_O and Na_2_EDTA at pH 4.2. On day 6, the root length was measured using a vernier caliper before returning seedlings to the solution containers with either control (pH 4.27) or the Al treatment solutions (pH 4.25) which contained 15 μM or 30 μM l Al^3+^ added as AlCl_3_.6H_2_O.

After 48 h in treatment solutions, the root length was measured again. The root tips (1−2 cm) were sampled using a scalpel blade, snap-frozen in liquid nitrogen, and stored in −80°C until RNA extraction. Three biological replicates were included for each line, with each replicate comprising 5 seedlings.

### Phylogeny development

The predicted amino acid sequences for the chickpea genome were downloaded from the NCBI database (BioProject: PRJNA190909, ASM33114v1 annotation Release 102). The MATE domain profile file (PF01554) was downloaded from the Pfam database^1^. The hmmscan program^2^ was used to identify the sequences containing the MATE domain. The amino acid sequences of previously reported MATE proteins were retrieved from the Uniprot database^3^. A list of previously characterized MATEs was retrieved from recent studies (Li et al., 2017; Wang et al., 2017). For phylogeny inference, amino acid sequence alignment was performed using MUSCLE (8 iterations) (Edgar, 2004). Phylogeny was developed using the Neighbor Joining (NJ) method implemented in MEGA 7.0 (Kumar et al., 2016) with the p-distance substitution model. A 1000 times bootstrap support was calculated for the developed NJ tree. Tree annotation was performed using the FigTree tool at http://tree.bio.ed.ac.uk/software/figtree/.

### Synteny and gene structural analyses

Synteny and gene duplication pattern were analyzed using MCScanX software (Wang et al., 2012). Chickpea genome annotation data were downloaded from the NCBI database (ASM33114v1, annotation Release 102). Intra- and inter-species genome comparisons were performed using the standalone NCBI-BLAST-2.2.29 tool with an E-value threshold of 1e-05, restricting the maximum hit number to 5. Collinear and tandem gene pairs were displayed using the family_tree_plotter tool in MCScanX package (Wang et al., 2012). Gene structure features were extracted from GFF file and were displayed using the GSDS 2.0 tool (Hu et al., 2015).

### Candidate multidrug and toxic compound extrusion genes retrieval and primer design

The amino acid sequences of *M. truncatula* MtMATE66 (GenBank ID AMP17768) and MtMATE69 (GenBank ID AMP17769) were used to query against the NCBI chickpea genome data using NCBI-BLAST-2.12.0+ (Altschul et al., 1990). The genomic DNA sequence and transcript sequence for the target MATE genes were retrieved. qRT-PCR primers spanning the introns were designed using the RealTime PCR Design Tool (Integrated DNA Technologies, United States^4^).

### RNA extraction and cDNA synthesis

The frozen root tips samples sampled from control and 15 μM Al^3+^ treatment were ground into a fine powder using a pestle and a mortar pre-cooled in liquid nitrogen. RNA extraction was carried out using Trisure^®^ (Bioline, Australia) by following the manufacturer’s instruction. ∼100 mg of ground tissue was used for each extraction. cDNA library construction was performed using SensiFAST™ cDNA Synthesis Kit (Bioline, Australia).

### qRT-PCR

The RT-qPCR experiments were carried out using SensiFAST™ SYBR No-ROX Kit (Bioline, Australia). Each reaction contains 5 μl SensiFAST mix, 4.2 μl cDNA template, 0.8 μl forward/reverse primers (500 nM). The RT-PCR primers are forward: CCTGCAGTGCTTCTCTCTTT and reverse: GCATACCCGGAAACTATGACA for *CaMATE1*, forward: GGCTTCCTTCAAGCTTCAATTC and reverse: GCAGGAGCACCAAATGATCTA for *CaMATE2*, forward: TACCCTCAGCGGAGCGAGC and reverse: GCTTTCAGCAACCAATTCTTTC for *CaMATE3*, and forward: AAGGAATTTTTCGCGGAATC and reverse: TGACTCCAAACCGGAATGTG for *CaMATE4*. RT-qPCR reaction was performed using the ViiA7 Real-Time PCR System (Thermo Fisher Scientific, United States) in 384-well plates. The previously tested chickpea CaCAC gene was used as a reference gene (Reddy et al., 2016). Three replicates were included for each sample. Each sample was run in three technical replicates. The transcription values were calculated using the comparative Ct method (2^–ΔCt^) (Schmittgen and Livak, 2008). The specificity of the primers were validated by melting curve analyses which showed a single clear peak.

### Carboxylic acid measurement

For CA measurements, chickpea seeds were germinated in a petri dish for 4 days and then transferred to hydroponic solution for 7 days growth. The growing conditions and nutrient solutions are the same as the root growth experiment described above. Two treatments: 0 μM Al^3+^ and 15 μM Al^3+^ were included. After cultivation, two or three seedlings for each line were transferred to a 50 ml tube with 0.2 mM CaCl_2_ solution (Pang et al., 2018). After growing for 60 min with aeration, 1-ml of the hydroponic solution was sampled and filtered through a 0.22 μm syringe filter into a HPLC vial. Three biological replicates were included for each line under both treatment conditions. The HPLC samples were acidified with a drop of orthophosphoric acid and frozen at −20°C until analysis. The HPLC analysis was carried out using previously described method (Cawthray, 2003). The dry weight of the chickpea root used for sampling was also measured after oven-dried at 60°C for 7 days.

### Transcriptional and genetic variation data mining

Chickpea gene expression atlas data published by Kudapa et al. (2018) was downloaded and screened for MATE-encoding genes. To match with the NCBI chickpea genome annotation, the amino acid sequences were used for blastp search. Only those genes with sequence identity > 95% were retained. The obtained transcriptional data in RPKM was scaled based on the maximum expression value for each individual gene. The transcriptional heat-map data was generated using the pheatmap R package (Kolde, 2015).

The genetic variation data for chickpea and wild *Cicer* lines was retrieved from the recently published chickpea pangenome (Varshney et al., 2021). The numbers of SNPs identified within the genetic regions of *CaMATE1-4* were counted based on their functional annotation.

### Protein modeling and substrate docking

Protein structural modeling was performed using the highly accurate Google AlphaFold tool (Jumper et al., 2021). The amino acid sequences for CaMATE2 and AtFRD3 were used as input for its online interface at https://colab.research.google.com/github/sokrypton/ColabFold/blob/main/AlphaFold2.ipynb. Five models were generated for each protein, with the top ranked model used for downstream analysis. Substrate binding cavity was predicted using CASTp tool (Tian et al., 2018). Small molecule docking was performed using the standalone Autodock Vina tool (Trott and Olson, 2010) (energy_range = 4 kcal/mol, exhaustiveness = 8). Docking files were prepared using MGLTools downloaded at https://ccsb.scripps.edu/mgltools/. Nine conformations were obtained for each receptor and ligand combination. Model visualizations were performed using PyMol (Schrödinger, LLC. Version 2.4.0) at http://www.pymol.org/pymol.

### Statistics analysis

Statistical differences for root length were tested by one-way ANOVA test. Gene transcriptional differences for qRT-PCR data were assessed using two-way t-tests.

## Results

### Effects of aluminum treatment on chickpea root growth

Eight wild *Cicer* germplasm lines, including 5 *C. reticulatum* and 3 *C. echinospermum* (Table 1), were used for Al tolerance assessment. In addition, one cultivated chickpea variety (PBA Pistol) was included as a reference. The wild *Cicer* accessions were selected based on a previous preliminary screening test (Vance et al., 2021), in which they displayed varied degrees of Al tolerance. In this study, the tolerance to Al toxicity was assessed by measuring the root elongation in solution culture under control (0 μM Al^3+^) and Al treatment (15 μM Al^3+^, 30 μM Al^3+^) conditions (Supplementary Table S1).

**TABLE 1:** Background information for chickpea accessions.

Collection sites	Name	AGG number	Type	Species	Genetic population
Bristepe2	Bari2_074	49809	wild	*C. reticulatum*	Ret_7
CudiB	CudiB_008B	49871	wild	*C. reticulatum*	Ret_11
Karatepe	Kayat_064	49969	wild	*C. reticulatum*	Ret_6
Sarikara	Sarik_067	50033	wild	*C. reticulatum*	Ret_6
Sarikara	Sarik_073	50035	wild	*C. reticulatum*	Ret_6
Destek	Deste_063	50109	wild	*C. echinospermum*	Ech_5
Destek	Deste_064	50111	wild	*C. echinospermum*	Ech_5
Karabahce	Karab_062	50140	wild	*C. echinospermum*	Ech_8
Australia	PBA_Pistol	PBA Pistol	cultivar	*C. arietinum*	NA

AGG stands for Australian Grains Genebank. Genetic population classification was based on a previous report (von Wettberg et al., 2018).

**FIGURE 1:**
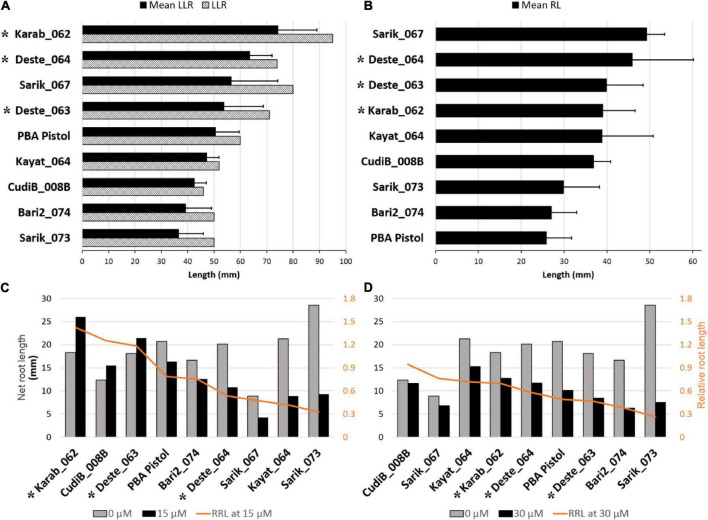
Root length under control and Al treatment conditions for target chickpea accessions. **(A)** Longest Length of Root (LLR) before treatment. The bar chart was sorted (highest to lowest) by mean LLR of different treatments for different accessions. **(B)** Mean Root Length (RL) before treatment. **(C)** Net root length at 15 μmol Al^3+^, and **(D)** 30 μmol Al^3+^ compared to the control condition (0 μmol Al^3+^). In graphs C and D relative root length (RRL) was calculated as: net root length at treatment/net root length at control. Plotting was sorted by RRL. (Error bar indicates standard deviation). * indicates the *C. echinospermum* lines.

Before applying the Al treatment (Figure 1A), the mean longest length of root (LLR) for the three conditions (each containing 10 seedlings) varied significantly (*p* = 0.0012), ranging from 37 mm (Sarik_073) to 74 mm (Karab_062). Notably, the mean LLR for the reference cultivar PBA Pistol, at 51 mm, ranked 5th among the 9 lines, supporting the use of PBA Pistol as a suitable reference for *Cicer* spp root growth assessment. The ranking of LLR for the nine *Cicer* spp lines is generally in agreement with the mean LLR (Figure 1A). Particularly, CudiB_008B was identified with the shortest LLR at 46 mm. When the mean root length (RL) was assessed, the target 9 nines also varied significantly (Figure 1B, *P* = 0.0057). The ranking is generally consistent with that for LLR, except that PBA Pistol was identified with the lowest mean RL. In addition, the 3 *C. echinospermum* lines (Deste_063, Deste_064, and Karab_062) were consistently ranked among the top 4 in terms of LLR and RL.

After growing in the treatment conditions for 48 h, change in length of the longest root (net root length) were measured for 15 μM Al^3+^ (Figure 1C), and 30 μM Al^3+^ (Figure 1D), and were compared to that under control condition. All except 3 wild *Cicer* lines (Karab_062, CudiB_008B, and Deste_063) displayed reduced root growth under both 15 μM Al^3+^ and 30 μM Al^3+^. The higher root growth of these 3 lines under 15 μM Al^3+^ than the control may be partly caused by the root length differences between 15 μM Al^3+^ and control before applying the treatment. The Al tolerance for each chickpea line was assessed by calculating the relative root length (RRL) (net root length growth at treatment/net root length growth at control). Based on RRL ranking, PBA Pistol was ranked as 4th and 6th at 15 μM Al^3+^ and 30 μM Al^3+^, respectively. Compared to the PBA Pistol, Karab_062 and CudiB_008B were consistently identified with higher RRL at both 15 μM Al^3+^ and 30 μM Al^3+^, suggesting they were Al tolerant genotypes. In contrast, Bari2_074 and Sarik_073 consistently displayed lower RRL, implying a potential Al sensitive genotype. In addition, Deste_063 displayed similar RRL with PBA Pistol under both Al treatments. Sarik_073 displayed the highest root growth under control but the lowest RRL at both 15 and 30 μM Al3 + treatments, suggesting this line as the most sensitive genotype. Finally, at 15 μM Al^3+^ treatment, the RRL for the three *C. echinospermum* lines were ranked medium to high among the 9 target lines. At 30 μM Al^3+^, these 3 lines displayed similar RRL with PBA Pistol, suggesting a medium Al tolerance.

### Identification of candidate multidrug and toxic compound extrusion transporter genes for aluminum tolerance

**FIGURE 2:**
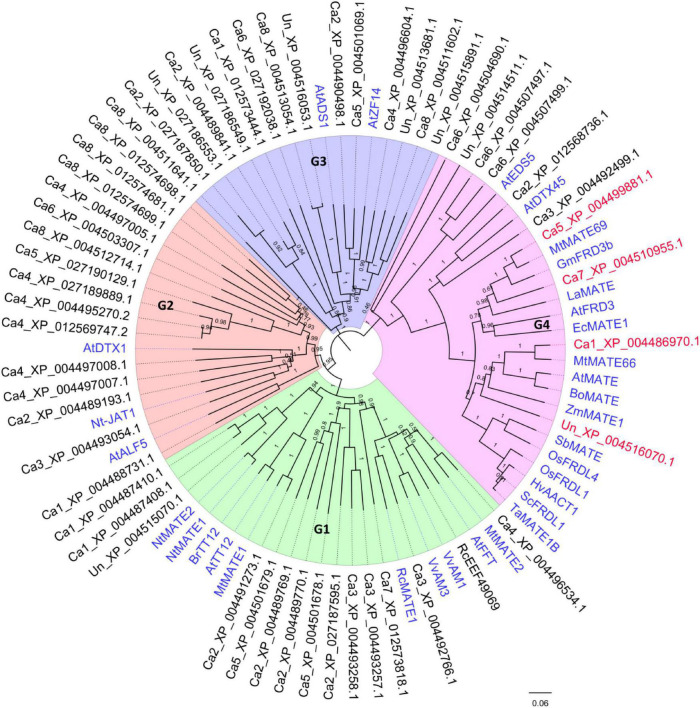
Phylogeny of MATE homologous genes in chickpea and other plants. The NJ phylogeny includes the chickpea protein sequences containing the MatE (PF01540) domain (retrieved from NCBI database BioProject: PRJNA190909). Previously characterized homologous MATE proteins were included as references (highlighted in blue). The target MATE genes *CaMATE1* and *CaMATE2* were in red. The Bootstrap support (1000 times iterations) was indicated above each branch.

Due to the potential involvement of MATE genes in Al-tolerance in plant, genome-wide identification of putative MATE genes in chickpea reference genome (NCBI BioProject: PRJNA190909) was performed. A total of 56 unique peptide sequences containing the MATE domain (Pfam ID: PF01554) were identified (Supplementary Table S2). To identify the candidate MATE genes for Al-tolerance in chickpea, a neighbor joining phylogeny was developed using previously characterized MATE-encoding genes as references. Out of the 56 MATE transporters identified, 2 partial proteins (<100 aa) were excluded from the phylogeny reconstruction. As shown in Figure 2, Chickpea MATE proteins generally divided into 4 major phylogenetic groups: G1-4. Specifically, 14 chickpea MATE genes were present in G1, which also contained some previously studied MATE-encoding genes *AtFFT* (Kitamura et al., 2016), *AtTT12* (Marinova et al., 2007), *BrTT12* (Chai et al., 2009), *MtMATE1* (Zhao and Dixon, 2009), *MtMATE2* (Zhao et al., 2011), *NtMATE1*, *NtMATE2* (Shoji et al., 2009), *VvAM1*, and *VvAM3* (Gomez et al., 2009). Most of these characterized MATEs were shown to be responsible for flavonoids transporting. Fifteen chickpea MATE genes were present in G2, together with *AtALF5* (Diener et al., 2001), *AtDTX1* (Li et al., 2002), and *Nt-JAT1* (Morita et al., 2009), which were suggested to confer toxin and heavy metal resistance. For G3, 15 chickpea MATE genes were present together with *AtADS1* (Sun et al., 2011) and *AtZF14* (Seo et al., 2012), which have multiple functions in disease resistance, organ initiation, Fe homeostasis, and hypocotyl cell elongation.

A total of 10 chickpea MATE genes were found in G4 (Figure 2). Within this group, 4 chickpea MATEs, including Ca5_XP_004499881 (CaMATE1), Ca7_XP_004510955 (CaMATE2), Ca1_XP_004486971 (CaMATE3), Un_XP_004516070 (CaMATE4), displayed close homology with previously characterized MATE genes responsible for citrate transportation in various plants, such as AtMATE and AtFRD3 (Rogers and Guerinot, 2002) in *Arabidopsis*, MtMATE66 and MtMATE69 (Wang et al., 2017) in *M. truncatula*, ZmMATE1 in maize (Maron et al., 2013), HvAACT1 in barley (Furukawa et al., 2007), and TaMATE1B in wheat (Tovkach et al., 2013). Most of these characterized MATEs have been shown as functional citrate transporters responsible for Al-tolerance, thereby supporting the potential involvement of *CaMATE1-4* in Al detoxification in chickpea. For the other 6 chickpea MATE genes in G4, Ca6_XP_004507497 and Ca6_XP_004507499 were clustered with AtEDS5 (Nawrath et al., 2002) of a disease resistance function, while Ca2_XP_012568736 was clustered with AtDTX45 (Tyra et al., 2007) of a plastid solute transportation function.

Based on their close homology with previously characterized citrate transporters in other plants, *CaMATE1-4* were selected as the potential candidate genes for Al-tolerance in chickpea. Gene annotation data suggest that *CaMATE1-4* were located on different chromosomes with the exception of *CaMATE4* whose chromosome location is unknown (Table 2). At the amino acid sequence level, using *M. truncatula* MATEs as the references (Wang et al., 2017), MtMATE69 has the highest similarity with CaMATE1 (89.07%), followed by CaMATE2 (68.21%), whilst MtMATE66 displayed the highest similarity with CaMATE3 (89.78%), followed by CaMATE4 (59.41%) (Table 2), which is consistent with their clustering patterns in the phylogenetic tree (Figure 2).

**TABLE 2:** List of homologous *MATE* genes identified in chickpea.

Gene ID	Chromosome	Location	Protein ID	Annotation	Identity with MtMATE69	Identity with MtMATE66
LOC101509308 (*CaMATE1*)	Ca5	NC_021164.1 (165223790.16529693)	XP_004499881.1	protein	89.07%	58.16%
LOC101514527 (*CaMATE2*)	Ca7	NC_021166.1 (397864610.39798690)	XP_004510955.1	DETOXIFICATION	68.21%	54.82%
LOC101497782 (*CaMATE3*)	Ca1	NC_021160.1 (111556850.11160403)	XP_004486970.1	43-like; MATE family;	61.69%	89.78%
LOC101509930 (*CaMATE4*)	Unknown	NW_004516700.1 (1435290.149043)	XP_004516070.1	TIGR00797	52.79%	59.41%

Gene annotation was based on NCBI genome assembly ASM33114v1. Amino acid sequence identity with M. truncatula MATEs was calculated.

### Synteny and gene structural analyses of multidrug and toxic compound extrusion genes

**FIGURE 3:**
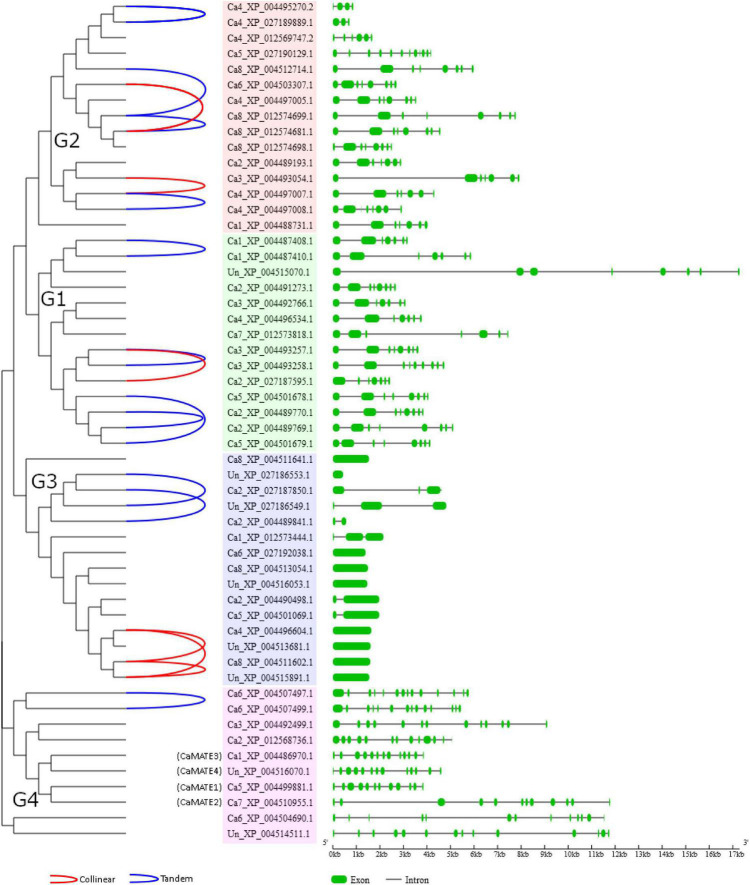
Synteny and gene structural analyses of chickpea MATE family. The synteny and gene structural features were displayed based on the developed MATE phylogeny. On the left, identified collinear and tandem duplication gene pairs were linked by red and blue lines, respectively. In the middle, phylogeny groups G1-G4 were highlighted in pink, blue, light green, and red, respectively. On the right, exon and intron features were displayed in green rectangle and black line, respectively.

Depending on the different genetic mechanisms, gene family expansion can be attributed to four gene duplication types: whole genome duplication (WGD)/segmental duplication, tandem duplication, proximal duplication and dispersed duplication. To further investigate the evolutionary relationship of the *Cicer* MATE genes, synteny and gene structural features were analyzed based on the developed phylogeny. As shown in Figure 3, a total of 6 collinear gene pairs and 11 tandem gene pairs (covering 27 genes) were identified for *Cicer* MATE genes, suggesting these genes have originated from WGD/segmental duplication and tandem duplication, respectively. The other chickpea MATE genes were classified as dispersed or proximal duplication. Specifically, *CaMATE1-4* were all identified as dispersed gene duplicates. Gene structural analyses showed that G1 and G2 MATE genes generally have similar exon-intron profiles, suggesting these two groups may have originated from a recent divergence event. In contrast, G3 and G4 displayed distinct gene structural profiles from G1 and G2. In particular, *CaMATE1* and *CaMATE2* contained 12 exons, whilst *CaMATE3* and *CaMATE4* had 13 exons, which is consistent with their phylogeny relationship. Despite their close homology, *CaMATE2* has clearly expanded intron regions compared to *CaMATE1*. Similarly, *CaMATE4* also clearly varied from *CaMATE3* in terms of intron length.

### *CaMATE2* and *CaMATE4* are highly transcribed in root tissues

**FIGURE 4:**
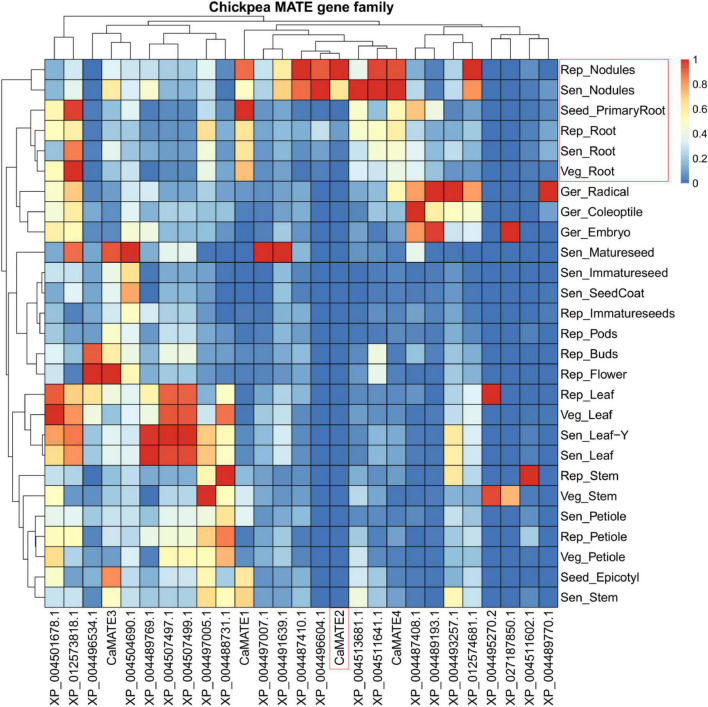
Transcriptional heat-map of *Cicer* MATE genes across different tissues. Transcriptional data for MATE domain containing genes in various tissues under different developmental stages were retrieved from the chickpea gene expression atlas and were scaled based on the maximum RPKM value of individual genes. Genes and tissues were clustered based on their expression profiles using pheatmap software (Kolde, 2015). Only those genes with matches in the NCBI annotation (ASM33114v1 annotation Release 102) were included. Root-specific *CaMATE2* and root-related tissues were highlighted in the red box.

To characterize the transcriptional profile of *Cicer* MATE genes, the transcriptional data for 27 MATE-encoding genes were identified from the chickpea gene expression atlas (Kudapa et al., 2018), which covers 27 tissues at 5 developmental stages: 24−48 h after imbibition, 8−10 days after germination (DAG), 25−30 DAG, 40−50 DAG, and 90−110 DAG (Figure 4). Overall, the transcription of MATE genes varies significantly in different tissues and developmental stages, displaying a clear tissue-specific and developmentally regulated pattern. Furthermore, the same tissue type seems to have coordinated expression of MATE genes at different developmental stages. For example, the root-relevant tissues (Rep_Nodules, Sen_Nodules, Rep_Root, Sen_Root, and Veg_Root) displays related MATE expression pattern and are clustered together in the heatmap. Similarly, seed-related tissues and leaf-tissues also form separate clusters. In addition, gene located on the left side of the heatmap tend to be expressed in multiple tissues and developmental stages, whereas those on the right are more tissue-specific at particular development period (Figure 4). Interestingly, the MATE gene clustering pattern based on expression profiles deviates greatly from the phylogeny classification pattern (Figure 4), suggesting that MATE genes within the same phylogeny group can have very divergent expression profiles.

Among the candidate MATE genes (*CaMATE1-4*) closely related to previously characterized citrate transporters, *CaMATE1* displays the highest expression in Seed_PrimaryRoot and is also highly expressed in other root and nodules tissues. In addition to root, *CaMATE1* also has abundant expression in Seed_Epitotyl and Sen_Stem, followed by Sen_Petiole and Veg_stem. In contrast to *CaMATE1*, the transcription of *CaMATE2* is highly root-specific, mainly transcribed in Rep_Nodules and Sen_Nodules. The absolute expression level of *CaMATE2* in root nodules is also much higher than that for other MATE genes (Supplementary Table S3). In addition to *CaMATE2*, *CaMATE4* also seems to be highly expressed in root tissues, except that moderate transcription of *CaMATE4* in various non-root tissues such as Ger_Radical, Ger_Coleoptile, Sen_Leaf, Sen_Leaf-Y, and Rep_Petiole was also observed. Lastly, *CaMATE3* has the most diverse expression pattern among these four MATE genes. It is commonly expressed in multiple tissues such as Rep_Flower, Sen_Matureseed, Seed_Epicotyl, Sen_Stem, Sen Nodules, Rep_Buds, and Rep_Pods at different developmental stages. Taken together, *CaMATE2* and *CaMATE4* were found to be highly expressed in root tissue, supporting their potential involvement in Al-tolerance.

### *CaMATE2* and *CaMATE4* are up-regulated under aluminum treatment in chickpea root tip

The MATE family genes encode transporter proteins that transport organic acid molecules, such as citrate or malate, from root to soil, thus facilitating the chelation of the toxic Al ions. The most active tissue in which the MATE genes are highly transcribed is the root tip (Takanashi et al., 2014).

**FIGURE 5:**
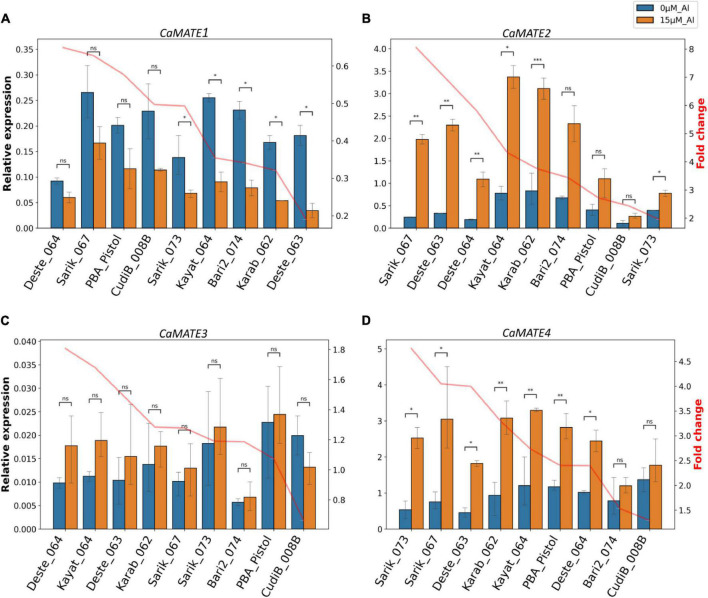
qRT-PCR analyses of chickpea MATE genes in root tips. The relative transcription of *CaMATE1* **(A)**, *CaMATE2* **(B)**, *CaMATE3* **(C)**, and *CaMATE4* **(D)** were determined in nine chickpea accessions at 0 and 15 μM Al^3+^ after 2 days of hydroponic cultivation. The previously determined *CaCAC* was used as the reference gene (Reddy et al., 2016). Transcription fold change (FC, in orange) at Al treatment condition compared to control was calculated. The plotting was sorted by FC from highest to lowest. *1.00e-02 < *p* < 5.00e-02, **1.00e-03 < *p* < 1.00e-02, ***1.00e-04 < *p* < 1.00e-03, n.s non-significant.

To explore the potential function of *Cicer* MATE genes in Al tolerance, the identified candidate genes *CaMATE1*-4 were selected for qRT-PCR experiments in root tips (1−2 cm) under control (0 μM Al^3+^) and treatment (15 μM Al^3+^). Results showed that the transcription of *CaMATE1* was consistently down-regulated upon Al treatment in all nine chickpea lines (Figure 5A). The fold change (FC) of *CaMATE1* transcription upon Al treatment ranged from 0.19 (Deste_063) to 0.65 (Deste_064). Both the most down-regulated line Deste_063 and the least down-regulated line Deste_064 belong to the *C. echinospermum* type. The other *C. echinospermum* line, Karab_062, displayed the second most down-regulated expression of *CaMATE1* (FC = 0.32) upon Al treatment. In addition, the expression of *CaMATE1* in PBA Pistol was only down-regulated moderately (FC = 0.58) upon Al treatment. In contrast to *CaMATE1*, *CaMATE2* was significantly up-regulated upon Al treatment in all target chickpea lines (Figure 5B). FC of *CaMATE2* upon Al treatment ranged from 1.95 (Sarik_073) to 8.05 (Sarik_067). The three *C. echinospermum* lines displayed relatively similar FC values upon Al treatment, with Deste_063 and Deste_064 ranked 2nd (FC = 6.93) and 3rd (FC = 5.80). Karat_064 and Karab_062 displayed the highest *CaMATE2* transcription at both control and Al treatment conditions. These two lines, together with Sarik_067 and Deste_063, had the highest FC and comparable *CaMATE2* transcription at 15 μM Al^3+^, implying the Al tolerant genotypes. The reference variety PBA Pistol displayed moderate *CaMATE2* expression at both conditions with FC = 2.72.

Compared to the control, no significant transcriptional change was observed for *CaMATE3* under Al treatment in all target chickpea lines (Figure 5C). Although there might be a variation in amplification efficiency for qRT-PCR primers, the relative expression level of *CaMATE3* to the reference gene appeared to be very low (Figure 5C), suggesting a weak transcription of *CaMATE3* in root tips. This observation is consistent with the transcriptome data in Figure 4, which also indicated that *CaMATE3* is weakly expressed in most root tissues. In contrast to *CaMATE3*, *CaMATE4* was up-regulated in all target chickpea lines upon Al treatment (Figure 5D), similar as that observed for *CaMATE2*. In terms of FC, Sarick_073 displayed the highest FC value for *CaMATE4* expression. This is in contrast with *CaMATE2*, which displayed the lowest FC for Saric_073 upon Al treatment. However, most chickpea lines including Sarik_067, Deste_063, Karab_062, Kayat_064, and CudiB_008B were ranked similarly in terms of FC for *CaMATE2* and *CaMATE4*’s responses to Al treatment. The abundant expression of *CaMATE2* and *CaMATE2* in root tips is consistent with the transcriptome data. This, together with their significant up-regulation upon Al treatment, support their potential involvement in Al-tolerance in chickpea.

### Chickpea root uses malonic acid as its main excreted organic acid

**FIGURE 6:**
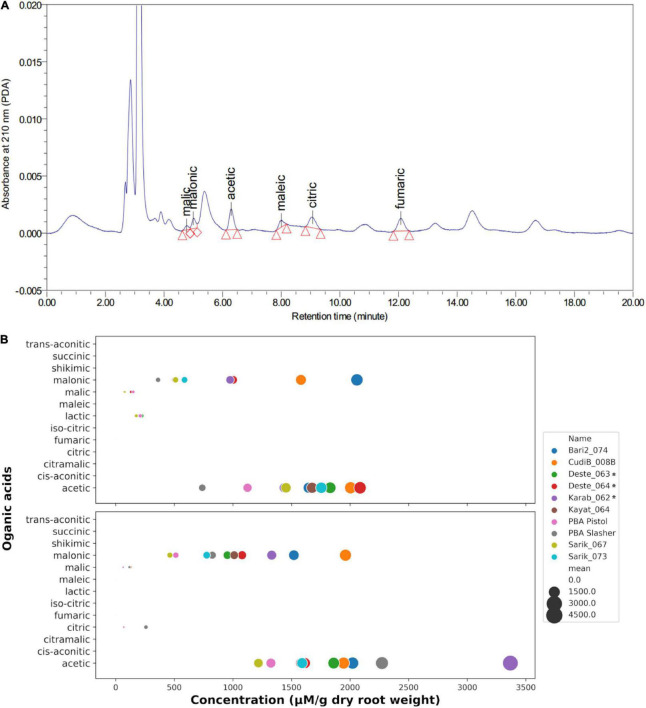
Determination of organic acids secreted by chickpea root tips. **(A)** A representative HPLC chromatography of organic acid (OA) measurement. **(B)** Quantification of the OA extrusion profiles in 10 target chickpea lines in control (upper panel) and Al treatment (lower panel).

To determine the organic acids secreted by chickpea root, a separate hydroponic experiment was performed for the target 9 *Cicer* lines together with chickpea cv. PBA Slasher. The hydroponic solutions under control and 15 μM Al^3+^ treatment were collected for organic acids profiling using reverse-phased column liquid chromatography approach. A total of thirteen common root organic acids (Supplementary Table S5) were tested. A representative chromatograph, displays the detection of two major organic acids: malonic acid and acetic acid (Figure 6A). In contrast, other acids were detected in low concentrations. After quantification, the contents of organic acids for each chickpea line under control and Al treatment were displayed in Figure 6B. Malonic acid and acetic acid were consistently identified as the major secreted acids under both control and treatment conditions. In addition, malic acid was also detected in both control and Al treatment conditions, albeit at much lower concentration and only occurring in some *Cicer* lines. Interestingly, a small amount of lactic acid was detected in four *Cicer* lines (Deste_063, PBA Pistol, Sarik_067, and CudiB_008B) under control, but none at the Al treatment condition. Furthermore, a small amount of citric acid was only detected in two cultivated chickpea (PBA Slasher, PBD Pistol) under Al treatment, but none under the control condition.

In terms of germplasm differences, the concentrations of the two major secreted acids varied greatly across different chickpea lines, suggesting an obvious genetic variation for organic acid excretion. The range of the malonic acid levels in the ten *Cicer* lines tends to be more stretched than that for acetic acid. At the control condition, Bari2_074, CudiB_008B, Deste_064, and Karab_062 produced the highest amount of malonic acid, ranging from 2058 to 976 μM per gram of dry root weight, whilst the remaining lines were similar in malonic secretion, ranging from 589 (Kayat_064) to 503 (PBA Pistol) with the exception of PBA Slasher, which produced the lowest malonic acid at 362 μM per gram of dry root weight. In contrast to malonic acid, the acetic acid levels did not vary too much for the top eight chickpea lines at control, ranging from 1439 to 2085 μM per gram of dry root weight. Similarly, the two cultivated chickpea PBA Slasher and PBA Pistol displayed the lowest acetic acid excretion.

Compared to the control, malonic acid secretion generally increased upon 15 μM Al treatment, with the exception of two chickpea lines Bari2_074 and Sarik_067. The highest increase in term of FC corresponds to PBA Slasher (FC = 2.28), Kayat_064 (1.72), and Deste_063 (1.62), followed by Karab_062 (1.36), Sarik_073 (1.32), and CudiB_008B (1.24), most of which overlapped with those lines displaying the highest CaMATE2 transcription at Al treatment with the exception of CudiB_008B. This observation supports a potential role of CaMATE2 in malonic acid secretion.

For acetic acid secretion, the FC under Al treatment varied greatly across the ten *Cicer* lines. PBA Slasher (FC = 3.08) and Karab_062 (2.34) displayed the highest increase in acetic acid excretion, followed by a slight increase in Bari2_074 (1.23) and PBA_Pistol (1.18). Another three lines Deste_063, CudiB_008B, and Kayat_064 displayed comparable or slight decrease in acetic acid extrusion under control and Al treatment, FC ranging from 1.02 to 0.94. In contrast, the remaining three lines, Sarik_073 (FC = 0.91), Sarik_067 (0.84), and Deste_064 (0.77), showed a moderate decrease in acetic acid excretion upon Al treatment.

In addition, malic acid excretion was detected in only three *Cicer* lines CudiB_008B, PBA Slasher, and PBA Pistol under both control and Al treatment. Significant increase of malic acid excretion upon Al treatment was observed in CudiB_008B (FC = 1.82) and PBA Slasher (1.50), whilst malic acid excretion decreased in PBA Pistol (0.42).

### *CaMATE2* displays varied substrate-binding potential

**FIGURE 7:**
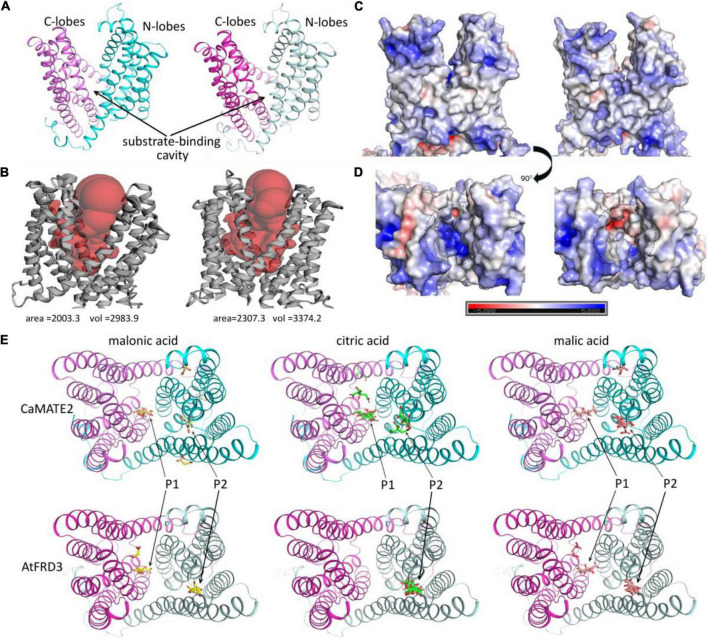
Protein structural modeling and substrate docking for CaMATE2 and AtFRD3. **(A)** Overall 3D structures of CaMATE2 (left) and AtFRD3 (right). The N-lobes and C-lobes were colored in cyan and pink, respectively. **(B)** Display substrate binding cavity. CaMATE2 (left) and AtFRD3 (right). **(C**,**D)** Display the electrostatic profiles. CaMATE2 (left) and AtFRD3 (right). **(E)** Substrate-docking of malonic acid (yellow), citric acid (green), and malic acid (red) to modeled CaMATE2 (top panel) and AtFRD3 (bottom panel). Positions P1 and P2 indicate two major substrate binding sites.

The finding that chickpea and wild Cicer root mainly secrets malonic acid, rather than citric acid or malic acid, is an interesting observation. To investigate its underlying molecular mechanisms, 3-dimensional protein structure models for CaMATE2 and AtFRD3 were generated and compared. As shown in Figure 7A, the overall structures of CaMATE2 and AtFRD3 are well conserved, each comprised of a N-terminal lobes and a C-terminal lobes. A cleft was formed between the two lobes as the putative substrate-binding sites. This substrate binding cavity was identified based on comparison with previously reported structures of MATE transporters (Zakrzewska et al., 2019). With a volume size of 2983.9 Å^3^, the substrate binding cavity of the CaMATE2 model is relatively smaller than that for AtFRD3 (3374.2 Å^3^) (Figure 7B). Similarly, the interior solvent-accessible area of the CaMATE2 cavity (2003.3 Å^2^) is also much smaller than that for AtFRD3 (2307.3 Å^2^). In terms of electrostatics, CaMATE2 and AtFRD3 displayed a similar profile in the exterior surface (Figure 7C). However, the charging profiles for their substrate binding cavities are clearly different (Figure 7D). Particularly, the substrate binding cavity for CaMATE2 is clearly more positively charged than that for AtFRD3, which may indicate different substrate-binding profiles for these two proteins.

To further investigate the substrate-binding specificity of CaMATE2 and AtFRD3, the small molecules of malonic acid, citric acid, and malic acid were docked to the protein models. For each ligand and receptor combination, the top nine binding conformations with the highest binding affinity were obtained and displayed in Figure 7E. For both CaMATE2 and AtFRD3, citric acid displayed the highest binding affinity, followed by malic acid and malonic acid, sequentially (Supplementary Table S6). The ranking of binding affinity for each acid is mainly determined by the number of carboxyl or hydroxyl groups. Overall, two major substrate-binding hotspots (Figure 7E, P1 and P2) can be identified. CaMATE2 and AtFRD3 displayed varied substrate binding preferences for different substrates. For AtFRD3, all of the nine binding-conformations of citric acid fell to P2. In contrast, malic acid and malonic acid can bind to both P1 and P2, but still with P2 as their preferred binding site. These observations suggest that AtFRD3 may use citric acid as its preferred substrate, consistent with its biological function (Durrett et al., 2007). In contrast to AtFRD3, both malonic acid and citric acid preferably binding to P1 in CaMATE2, whilst malic acid preferably binding to P2 in CaMATE2 (Figure 7E and Supplementary Table S6). In addition, the top nine conformations for all of the three acids were distributed between P1 and P2 in CaMATE2. These observations revealed a clear difference between the substrate binding cavities for CaMATE2 and AtFRD3. Whilst the preferential binding substrate for CaMATE2 cannot be definitively determined based on the substrate docking, the lack of a clear binding formation for citric acid in CaMATE2, in contrast to that in ArFRD3, may indicate a clear shift in their substrate binding specificity.

### Wild *Cicer* has much higher genetic diversity than cultivated chickpea for *CaMATE2* and *CaMATE4*

To assess the genetic diversity of *CaMATE1-4* in chickpea germplasm, the recently published chickpea pangenome (Varshney et al., 2021) was searched for SNPs occurring in the target genes (Table 3). The pangenome data covers 3171 cultivated chickpea and 195 wild *Cicer* lines. As shown in Table 3, despite of a much smaller population, wild *Cicer* displayed much higher levels of SNPs than chickpea cultivars for all of *CaMATE1-4*. This implies a much higher genetic diversity within wild *Cicer* lines. Particularly, *CaMATE1*-4 are strictly conserved in chickpea cultivars, with only 2 ∼ 5 non-synonymous SNP mutations, respectively. In contrast, 30 ∼ 55 non-synonymous SNPs were identified in the 195 wild lines. After taking into consideration the length of CDS sequences, the levels of non-synonymous mutations were similar for *CaMATE2-4*, except CaMATE1 for which the level of non-synonymous mutations was relatively higher. For the putative regulatory regions (Up_1000 bp and Down_1000 bp), chickpea cultivars also displayed very limited variations for both genes. For the Up_1000 bp region, *CaMATE3-4* had relatively more SNPs than *CaMATE1-2*. For the Down_1000 bp region, *CaMATE4* displayed relatively more SNPs than *CaMATE1-3*. Taken together, genetic variation analyses revealed a much higher genetic diversity in wild *Cicer* lines for the target genes, which supports wild *Cicer* lines as an effective genetic pool for identifying tolerant genotype of Al stress.

**TABLE 3:** Number of SNPs identified in *CaMATE1* and CaMATE2.

SNPs	*CaMATE1* (CDS = 1617 bp; Intron = 2239 bp)		*CaMATE2* (CDS = 1257 bp; Intron = 4415 bp)		*CaMATE3* (CDS = 1521 bp; Intron = 2350 bp)		*CaMATE4* (CDS = 1452 bp; Intron = 2845 bp)	
				
	Cultivated	Wild	Cultivated	Wild	Cultivated	Wild	Cultivated	Wild
Up_1000 bp	2	51	9	59	6	90	1	87
Down_1000 bp	0	52	3	46	3	48	8	86
Synonymous	0	36	1	58	7	60	3	56
Non-synonymous	2	55	3	30	5	34	4	33
Intron	11	216	15	211	30	262	15	288

SNPs were mined from the chickpea pangenome resequencing data. The number of SNPs was calculated based on different SNP effects and chickpea lines. Up_1000 bp and Down_1000 bp refer to genetic regions 1000 bp upstream of start codon and 1000 bp downstream of stop codon, respectively.

## Discussion

Our results showed that there was significant variation in Al-tolerance among the target wild *Cicer* lines, thereby supporting the potential use of wild *Cicer* for Al-tolerance improvement in chickpea breeding. Using the commercial chickpea cultivar PBA Pistol as a reference, the target wild *Cicer* accessions displayed both tolerant and sensitive phenotypes. Indeed, genetic variation analyses in the chickpea pangenome showed that wild *Cicer* displayed much higher genetic diversity than chickpea cultivars. Compared to other species, chickpea is relatively susceptible to Al-stress (Singh et al., 2012; Choudhury and Sharma, 2014). To date, two studies have attempted to examine the genotypic variations against Al-stress. The assessment of Al-tolerance in 35 and 24 cultivated chickpea genotypes, respectively, have allowed the identification of relatively tolerant and sensitive chickpea lines (Singh and Raje, 2011; Manorma Sharma et al., 2015). These Al-tolerant lines may be used for yield improvement in chickpea breeding. However, compared to the other crop species, the genetic diversity of chickpea germplasm against various other abiotic and biotic stresses is also relatively narrow (Berger et al., 2003; Singh et al., 2008), which hinders the progress on chickpea breeding toward higher yield under unfavorable environmental conditions. The lack of sufficient genetic diversity in chickpea, however, can be complemented by some of its wild progenitors such as *C. reticulatum* and *Cicer echinospermum*, which display no reproductive barrier with cultivated chickpea (Pundir and Van Der Maesen, 1983; Singh et al., 2008). Based on these observations, the current study attempted to evaluate the Al-tolerance variation in these two species.

The Al-activated MATE transporter facilitates the secretion of citrate from the root apex, which is the major mechanism of Al-tolerance in many plants (Bojorquez-Quintal et al., 2017). The availability of the chickpea genomic data (Varshney et al., 2013) has enabled the genome-wide survey of putative MATE-encoding genes in the present study. Based on the most recent chickpea genome annotation, we identified a total of 56 MATE homologs in *Cicer*, which is close to the 71 reported for *Populus* (Li et al., 2017) but significantly less than the 117 for soybean (Liu et al., 2016). Phylogeny analysis suggested that the MATE gene family could be divided into four major subclades, which is similar with the observation made in other species such as soybean (Liu et al., 2016) and *Populus* (Li et al., 2017). In our phylogeny, 4 *Cicer* MATE homologs were clustered each with the previously identified AtMATE (Liu et al., 2009) and AtFRD3 (Durrett et al., 2007), respectively, which resembled the observation in *Populus* (Li et al., 2017). In contrast, the soybean reference genome contained 4 close homologs each for AtMATE and AtFRD3, respectively (Liu et al., 2016), which may result from its recent polyploidy.

Based on the assessment of Al-sensitivity in the progeny of two chickpea parental lines, Singh and Raje (2011) determined that the Al-tolerance variation in the two parental lines may be controlled by a single dominant gene. However, the underlying candidate gene and its physiological mechanism were not identified. In this study, we identified *CaMATE1-4* as the initial candidates for Al-tolerance in chickpea based on phylogeny clustering and homology search. Furthermore, we found that *CaMATE2* and *CaMATE4* were significantly up-regulated upon Al treatment in the root tips of most chickpea lines, supporting these 2 genes’ potential involvement in Al-tolerance in chickpea. The abundant expression of *CaMATE2* and *CaMATE4* in root tips based on RT-PCR data is also in a good agreement with the transcriptome data. Future study is necessary to verify if *CaMATE2* or *CaMATE4* may underlie the previously reported genetic locus for Al-tolerance in chickpea (Singh and Raje, 2011). In the model legume species *M. truncatula*, MtMATE66 and MtMATE69 have been identified as effective citric acid transporter in the root tissue and both genes have been shown to be induced by Al treatment (Wang et al., 2017). In our phylogeny, CaMATE1 and CaMATE3 displayed the highest similarity with MtMATE69 and MtMATE66, respectively. However, the transcription of *CaMATE1* and *CaMATE3* were found non-responsive to Al-treatment. Instead, *CaMATE2* and *CaMATE4* were clearly induced by Al treatment, suggesting a potential species-specific gene evolution. To gain insights on the biological role of *CaMATE1* and *CaMATE3* in chickpea, further study is needed to explore their complete transcriptional profiles in other tissues or developmental stages. Al-inducible MATE citrate transporters have been reported for *AtMATE* in *Arabidopsis* (Liu et al., 2009), *OsFRDL4* in rice (Yokosho et al., 2011), *ZmMATE1* in maize (Maron et al., 2013), SbMATE in sorghum (Magalhaes et al., 2007), *GmMATE75* in soybean (Liu et al., 2016), *HvAACT1* in barley (Furukawa et al., 2007), and *PtrMATE1*, *PrtMATE2*, *PtrDXT2*, and *PtrDXT27* in *Populus* (Li et al., 2017). All of these Al-induced MATE genes have been shown to be involved in Al-tolerance. In *Arabidopsis*, both AtFRD3 (Durrett et al., 2007) and AtMATE (Liu et al., 2009) have been identified as functional citrate transporters and were both shown to confer Al-tolerance. Similar observations have been made for MtMATE69 and MtMATE66 (Jaiswal et al., 2018). However, the mechanisms of Al-tolerance associated with these two groups of genes tend to vary. AtFRD3 is responsible for citrate and iron translocation in the xylem tissues (Durrett et al., 2007), while AtMATE functions for citrate extrusion in the root tips (Liu et al., 2009). These different biological functions were presumed to be caused by their transcription in different tissues (Liu et al., 2009). Consistent with their biological function, the transcription of *AtMATE* is induced by Al treatment, whereas *AtFRD3* is not responsive to Al (Liu et al., 2009). In contrast to *AtFRD3*, *MtMATE69* appeared to be induced by both Al-treatment and Fe-deficiency (Wang et al., 2017; Jaiswal et al., 2018). In addition to their function in Al-tolerance, *AtFRD3* (Durrett et al., 2007) and *MtMATE69* (Wang et al., 2017), which belonged to the same cluster with *CaMATE1* and *CaMATE2*, and functioned in the transportation of citrate to xylem, have been shown to be functional in iron homeostasis. It remains to be determined if this function is conserved for *CaMATE1* and *CaMATE2* or not.

In our qRT-PCR analyses, the levels of *CaMATE2* and *CaMATE4* expression varied greatly among the target nine *Cicer* lines, suggesting a potential genetic variation in Al-tolerance. Indeed, genetic variation also revealed a much higher number of SNPs in the wild *Cicer* lines. These observations, together with the root elongation assessment, provide further support that wild *Cicer* lines contain a more diverse genetic pool for Al-tolerance. Consistent with their potential role in Al-tolerance, *CaMATE2* and *CaMATE4* displayed relatively higher transcription in the chickpea accessions which generally ranked higher in the assessment based on root growth. In barley, Al-tolerant varieties displayed significantly longer root elongation than Al-sensitive lines, which is associated with higher *HvAACT1* transcription in the root tips (Fujii et al., 2012). It would be intriguing in further study to identify superior alleles of *CaMATE2* and *CaMATE4* that are associated with Al-tolerance and can be used in chickpea breeding. In addition to the SNPs, future study can also be devoted to identify other types of genetic polymorphism such as insertion or deletion.

It should be noted that some chickpea accession included in this study, such as CudiB_008B, displayed the lowest *CaMATE2* and *CaMATE4* transcriptions but was ranked as Al-tolerant based on root elongation assessment. This suggests that Al-tolerance in chickpea may be controlled by multiple genes and pathways. In addition to citrate transporters, malate transporters that confer Al-tolerance in plants have also been characterized in *Arabidopsis* (*AtALMT1*) (Hoekenga et al., 2006) and wheat (*TaAMLT1*) (Sasaki et al., 2004), both of which are induced by Al treatment. The corresponding homologous genes in chickpea remain to be determined and investigated. Comprehensive transcriptome profiling in medicago and soybean root tips have revealed that many genes related to oxidative stress, transcriptional regulation, cell wall process, lignin deposition are also responsive to Al treatment (Chandran et al., 2008; You et al., 2011). Comparative transcriptome study is also necessary to unravel other potential genetic mechanisms associated with Al-tolerance in chickpea. Transgenic over-expression of *ALMT* homologs in medicago and soybean have also been shown to increase Al-tolerance (Chen et al., 2013; Liang et al., 2013). In *Arabidopsis*, AtSTOP1, a C2H2 zinc finger transcription factor that regulates the expression of AtMATE and AtMLT1, is also involved in Al-tolerance (Iuchi et al., 2007). The AtSTOP ortholog in rice, OsART1, has also been characterized to be related to Al-tolerance (Yamaji et al., 2009). Recently, the effect of microRNAs on Al-tolerance in barley was tentatively investigated, providing new insights into this complex biological process (Wu et al., 2018). Therefore, it is necessary for future study to verify if a similar genetic basis for controlling Al-tolerance may be present in chickpea or not. On another note, legume plants including chickpea can characteristically form nodules in the root for N-fixation. Aluminum in acidic soils may pose an additional constraint on nodule-function due to the inhibition of nodule formation or on rhizobia, *per se* (Jaiswal et al., 2018). As an earlier study has shown, most acid-tolerant chickpea mesorhizobia showed transcriptional induction of major chaperone genes upon acid treatment, whilst the sensitive strains showed repression (Brigido and Oliveira, 2013). In addition, for the improvement in chickpea production in acidic soil, attention should also be given to manganese toxicity tolerance (Pradeep et al., 2020).

Citrate and malate are the most common CAs extruded by plant roots. In wheat seedlings (Ryan et al., 2009), the highest secreted organic acid corresponds to citrate, followed by malate. In *Arabidopsis* (Liu et al., 2009), however, a higher level of malate is produced than citrate. Interestingly, in this study, we determined that malonic acid is the major secreted acid by chickpea root. Our observation is corroborated by an earlier reports (Veneklaas et al., 2003) which also detected malonate as the major extruded carboxylate in the rhizosphere of chickpea and lupin (*Lupinus perennis*) in soils. In the studies of Veneklaas et al. (2003), citrate was also constantly detected at considerable levels, followed by malate, albeit at much lower levels, while Kabir (Kabir et al., 2015) found similar levels of citrate and malate in root rhizosphere. In contrast, our study only found a small amount of malic acid and citric acid in some chickpea accessions. These variations may be caused by the differences in CA excretion under soil and hydroponics conditions. In addition, the short incubation time (60 min) in our CA sampling may also be contributing factor. In addition to malonic acid, we also detected acetic acid at abundant levels in the hydroponic solutions for all chickpea accessions. However, the excretion of acetic acid may not be related to Al treatment because there was no clear pattern between control and treatment. In contrast, the levels of malonic acid generally increased upon Al treatment for most chickpea accessions included in this study. The utilization of malonic acid as the major extruded CA may be explained by the different substrate-binding cavity in CA transporters, which is exemplified in our protein structural modeling and substrate docking analyses for CaMATE2. In addition to CaMATE2, the substrate specificity of CaMATE4 should also be investigated in future study, which may also contribute to malonic acid secretion. Future in-depth protein functional analyses are needed to verify our hypothesis. On the other hand, the use of malonic acid as its major secreted acid may also explain its relatively Al-sensitive phenotype compared to other species. This is due to the fact that the Al-chelating capacity of organic acids varies (citrate > oxalate > malate) (Li et al., 2009). Based on the molecule structure, the Al-chelating capacity of malonic acid may be higher than malate but lower than citrate and oxalate. Therefore, future genetic engineering of Al-tolerance in chickpea may also aim to modify chickpea genes to excrete citrate.

## Conclusion

We assessed and verified the presence of significant Al-tolerance variation across 8 wild *Cicer* genotypes. We identified *CaMATE2* and *CaMATE4* encoding putative organic acid transporters that were abundantly transcribed and significantly up-regulated under Al-stress in chickpea root tips, representing the potential candidate genes related to Al-tolerance in chickpea. We found that chickpea root mainly excretes malonic acid, which may be related to a potential difference in the substrate-binding specificity of its CA transporters.

## Data availability statement

The datasets presented in this study can be found in online repositories. The names of the repository/repositories and accession number(s) can be found in the article/Supplementary material.

## Author contributions

CL, RB, and WV supervised the study. YJ wrote the manuscript. KP, YJ, XZ, WV, HW, and ZD performed the hydroponic tests. YJ, BW, XZ, HW, and ZD did the qRT-PCR experiments. JB provided the chickpea materials and data analysis. XZ, XX, and CZ assisted the laboratory experiments. YJ performed the bioinformatics and data analyses. JB, RB, CL, and WV provided the valuable revisions. All authors have read the manuscript.

## Footnotes

1
https://pfam.xfam.org/

2
http://hmmer.org/

3
https://www.uniprot.org/

4
https://sg.idtdna.com/scitools/Applications/RealTimePCR
